# A Coronary Cameral Fistula Associated With Incessant Ventricular Arrhythmias

**DOI:** 10.7759/cureus.35847

**Published:** 2023-03-06

**Authors:** Ghulam Mujtaba Ghumman, Amir Khan, Madeeha Shafqat, Jay Shah, Hemindermeet Singh

**Affiliations:** 1 Internal Medicine, St. Vincent Mercy Medical Center, Toledo, USA; 2 Cardiology, St. Vincent Mercy Medical Center, Toledo, USA; 3 Interventional Cardiology, St. Vincent Mercy Medical Center, Toledo, USA

**Keywords:** torsade de pointes, ventricular, arrhythmias, cardiac chamber, cameral, fistula

## Abstract

Coronary cameral fistula (CCF) is a rare congenital abnormality of abnormal communication between coronary arteries and cardiac chambers. Most patients are asymptomatic, while symptomatic patients are usually present in childhood. Adult patients can present with angina, heart failure, or arrhythmias. We report a rare case of CCF with associated recurrent torsade de pointes (TdP) in the absence of ischemic heart disease and electrolyte abnormalities.

## Introduction

Coronary cameral fistula (CCF) is an abnormal communication between coronary arteries and cardiac chambers [1]. It is reported in less than 1% of the population and can be seen in 0.1-0.2% of coronary angiographies [2]. The most common fistulous connection occurs between the right coronary artery and right ventricle, but it can also originate from the left coronary system and drain into the left heart chambers [3]. Most cases of CCF are asymptomatic. Adult patients with large fistulas, classically described as >250 mm, can present with angina, heart failure, or arrhythmias [4,5]. We report a case of large CCF originating from the right posterior descending coronary artery (RPDA) and draining into the right ventricle that presented with recurrent torsade de pointes (TdP).

## Case presentation

**Figure 1:**
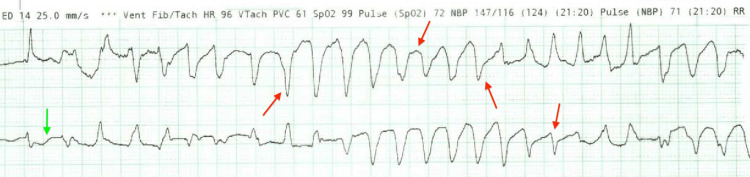
Continuous telemetry rhythm strips showing torsade de pointes (red arrows) and prolonged QT (green arrow) The image shows polymorphic ventricular tachycardia with gradual change in amplitude and twisting of the QRS complexes around the isoelectric line (red arrows) along with the prolonged QT (green arrow).

A 73-year-old female presented after her automated implantable cardioverter defibrillator (AICD) device fired multiple times. Her pertinent history included chronic heart failure with reduced ejection fraction secondary to non-ischemic cardiomyopathy and the implantation of a subcutaneous AICD post-ventricular fibrillation arrest. She has had no chest pain, shortness of breath, or lower extremity edema. Vital signs on arrival showed blood pressure of 120/99 mmHg, a pulse of 87 beats per minute, a respiratory rate of 22 breaths per minute, and a good saturation on room air. There was no murmur, lung crepitations, or jugular venous distention. The ECG showed normal sinus rhythm with occasional premature ventricular complexes. Potassium was 3.1 and magnesium was 1.8 on admission. Continuous telemetry revealed recurrent episodes of ventricular tachycardia (VT) storm in the form of TdP (Figure 1).

**Figure 2:**
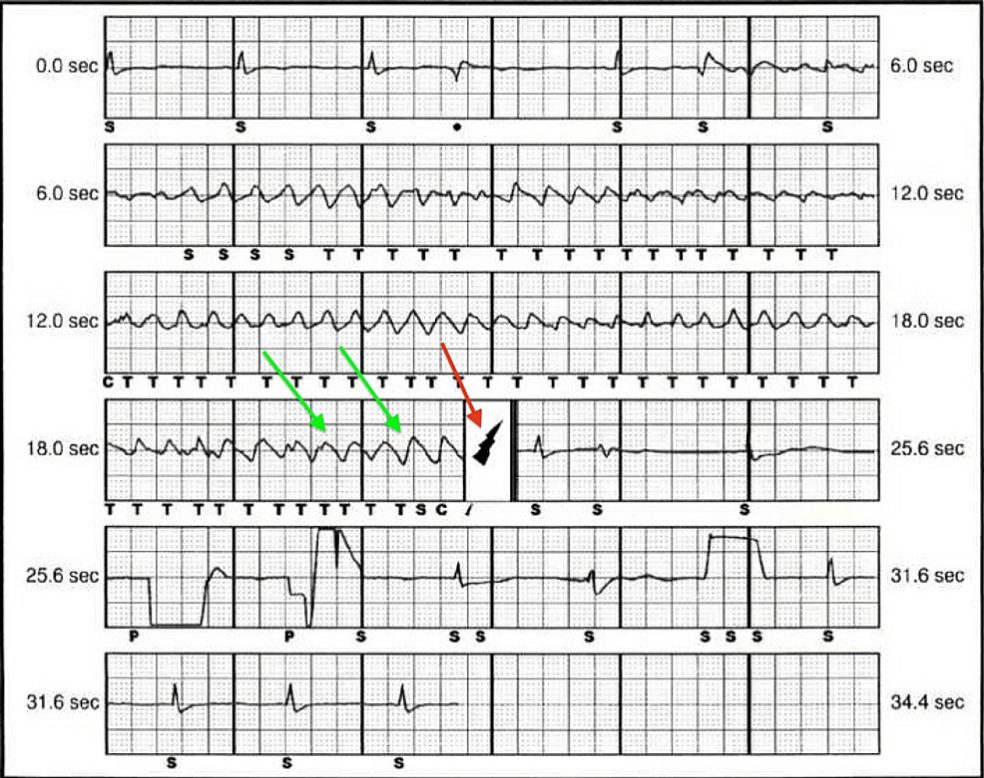
AICD (automated implantable cardioverter defibrillator) interrogation showing torsade de pointes (green arrows) with appropriate defibrillation by AICD (red arrow)

She continued to have polymorphic VT despite the aggressive replacement of potassium and magnesium, with subsequent levels in the normal range. The patient was started on amiodarone infusion and later switched to lidocaine due to prolonged QTc. AICD interrogation showed TdP with multiple appropriate defibrillations (Figure 2).

A transthoracic echocardiogram showed an ejection fraction of 35-40% with hypokinesis of the apical and anteroseptal walls. A repeat coronary angiography was obtained due to recurrent arrhythmias and a decline in left ventricular ejection fraction (LVEF), which showed normal coronaries; however, there was a large CCF connecting the mid-RPDA to the right ventricle (Video 1).

**Video 1:** Coronary angiography showing a large-sized coronary cameral fistula between the right posterior descending coronary artery and right ventricle

This finding of a large fistula was also seen on her previous angiogram around three years ago. The patient was continued on a lidocaine infusion with suppression of VT; the drip was later weaned off. The patient was transitioned to metoprolol succinate. She was discharged home in stable condition.

A few days later, the patient returned with a similar presentation of ICD shock, with device interrogation showing a VT storm. The patient was stabilized and then transferred to a tertiary care facility for consideration of VT ablation and embolization of the CCF. At the tertiary care facility, a cardiac nuclear medicine PET (positron emission tomography) scan was done that did not show any abnormality other than an ejection fraction of 43%. The patient continued to have further episodes of VT, prompting an electrophysiology study with radiofrequency ablation of the posterior right ventricular outflow tract and papillary muscle ectopy. The patient was subsequently maintained on mexiletine. Follow-up of the latest records showed a recurrence of ventricular arrhythmias even after radiofrequency ablation.

## Discussion

CCF are rare congenital anomalous communications that can be single or multiple and occur between one or more coronary arteries and cardiac chambers due to deviations from normal embryological development. It may also be acquired from trauma (stab, gunshot, or projectile injuries) or invasive heart procedures such as coronary angiography or pacemaker placement [6].

Most CCF patients are asymptomatic, while the most common presentation in symptomatic patients includes chest pain or heart failure; however, arrhythmias are rarely associated. A review study on coronary artery fistulas showed that 55% of patients were asymptomatic, 34% had angina, and 13% had heart failure [6].

Typically, blood flow follows a less resistant pathway, but when the fistula site of drainage is in a left cardiac chamber, it creates a left-to-left shunt, mimicking aortic valve regurgitation, resulting in volume overload on the left ventricle [7]. CCFs that drain to the right heart chambers follow the less-resistance pathway of the pulmonary circuit, as seen in our case. It is important to remember that the coronary to pulmonary artery fistulas will have similar functional significance and mostly drain into the main pulmonary trunk [8]. Our patient had an anatomic type of CCF, which can potentially lead to the coronary steal phenomenon. A large fistula pulls blood away from a normal coronary pathway with widened pulse pressure and subsequent demand ischemia and LV dysfunction, which ultimately can lead to a nidus for ventricular arrhythmias. A case report has described exercise-induced ventricular arrhythmia, which resolved after surgical ligation of the fistula [9]. Another case has been reported of atrial fibrillation associated with multiple coronary cameral fistulas, where medical management with amiodarone and subsequent metoprolol led to the resolution of symptoms [10].

Some patients with CCF can have a murmur that is loud and continuous at the lower or midsternal border, depending upon the drainage site. Coronary angiography remains the definitive diagnostic tool. Computed tomography coronary angiography (CTCA), magnetic resonance imaging (MRI), and transesophageal echocardiography (TEE) are also being utilized to diagnose CCF [11,12]. The management of CCFs depends on the symptoms and size of the fistula. There are no clear guidelines or expert consensus given the condition’s rarity. Asymptomatic patients with small fistulas can be observed with close follow-up [13].

Treatment options for symptomatic patients with large fistulas include surgical ligation or percutaneous transcatheter closure. Although surgical obliteration of the fistula is the most effective treatment, both techniques lead to a good prognosis. It is important to continue antiplatelet therapy after the closure of the fistula [14,15]. Betablocker use has been described in patients with fistulas not amenable to surgery, especially in patients presenting with arrhythmias [16].

## Conclusions

Coronary Cameral fistula is a relatively uncommon form of coronary artery fistula. Most of these fistulas are asymptomatic, especially if they are small. Large fistulas can present with chest pain or heart failure; however, arrhythmias are rare. Symptomatic patients with large fistulas should undergo fistula closure. In our case, the large coronary cameral fistula presented with incessant/recurrent ventricular arrhythmias, and the patient would benefit from surgical or percutaneous closure for definitive management. Our case adds to the literature and highlights the relatively rare presentation of coronary cameral fistulas with polymorphic ventricular arrhythmia.
